# A Critical Review on BH3 Mimetic Drugs and the Treatment of Cancer‐Associated Thrombosis (CAT): A Proposed Design for a Drug Delivery System Capable of Simultaneously Targeting Tumor Cells and Activated Platelets

**DOI:** 10.1002/cam4.71270

**Published:** 2025-09-19

**Authors:** Mehran Ghasemzadeh, Nazanin Heidari, Jalal Naghinezhad, Alireza Ghasemzadeh, Ehteramolsadat Hosseini

**Affiliations:** ^1^ Blood Transfusion Research Center, High Institute for Research and Education in Transfusion Medicine Tehran Iran; ^2^ School of Medicine Iran University of Medical Sciences Tehran Iran

**Keywords:** apoptosis, BH3 mimetic, cancer, drug delivery system, nanoparticle, necrosis platelet, RGD, thrombocytopenia, thrombosis

## Abstract

**Background:**

Induction of programmed cell demise against tumors that achieves selective targeting of the cancerous state without side effects on healthy tissues and cells is the most challenging therapeutic goal to eradicate cancer progression. In this regard, several BH3‐mimetic drugs have been designed to induce apoptosis in cancer cells with acceptable specificity and fewer adverse events.

**Implications:**

Taking all considerations into account, even the latest versions of ‐BH3 mimetics or some other systemic anticancer drugs may affect platelets, mainly manifested by thrombocytopenia in cancer patients who are per se at major risk of hemostatic complications. This is mainly due to the fact that platelets, as anucleated cells, are more vulnerable to apoptosis, especially induced by earlier versions of BH3‐mimetics. On the other hand, the cancerous state, particularly in its aggressive conditions, is usually associated with the risk of thrombosis and thromboembolism. Therefore, given that some earlier versions of BH3‐mimetics have the potential to simultaneously damage platelets and cancer cells, they may be considered as a therapeutic choice for the treatment of cancer‐associated thrombosis (CAT). However, this is subject to the design of a specific platform of drug carriers that supports cancer targeting without interfering with other tissues and cells. The critical review presented here first provides an overview of the various BH3‐mimetic drugs available, highlighting ongoing development to enhance their safety and efficacy. Then, by introducing studies on the direct delivery of BH3‐mimetics, this review finally proposes an innovative approach for the “conserved conveyance” of drugs to effectively cotarget cancer cells and activated platelets at the site of CAT.

**Conclusion:**

Notably, the main advantage of the proposed drug delivery system presented here is its minimal interference with natural hemostasis, where the drug is expected to attack only tumor cells and CAT, without affecting circulating platelets required for physiological thrombus formation and proper hemostasis.

## Graphical abstract

Targeting Cancer associated thrombosis (CAT): The left panel (A) shows cancer‐associated thrombosis with activated platelets expressing α_IIb_β_3_ in close contact with tumoral vasculature expressing α_v_β_3_ integrin, where both cells release a significant amount of thrombin. In the middle panel (1–8), the suggested design for RGD‐decorated nano‐particle has been described. Here, a secondary nano‐particle (3) consists of a liposomal core that is loaded with BH3‐mimetics (1) and coated with c‐RGD (2). The secondary nano‐particle is then enclosed with thrombin‐sensitive linkers (4) in an albumin cage (5), resulting in the formation of a primary nano‐particle (6) which is subsequently functionalized with i‐RGD (7), that converts it to the primary nano‐particle (8). As shown in the magnified image (B), the primary nano‐particle specifically recognizes the α_v_β_3_ integrin expressed by tumor cells, where, in the presence of thrombin, the thrombin‐sensitive linkers are cleaved, releasing the secondary nano‐particle into the metastatic cancerous site. The secondary nano‐particle can then recognize either activated platelet or tumor cell integrins through their c‐RGD. Upon binding to the cancer or activated platelet, the BH3‐mimetics enter the cytoplasm of the tumor cells or activated platelet, triggering the apoptotic pathway (C).

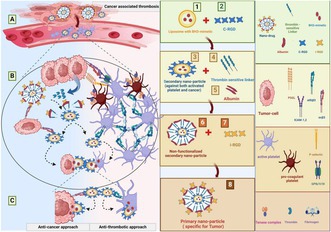

**Abbreviations**
ADAMs: A disintegrin and metalloproteinase
ANT: adenine nucleotide translocator
ATP: adenosine triphosphate
BAD: BCL2 associated agonist of cell death
BAK: Bcl‐2 antagonist/killer
BAX: BCL2 Associated X
BCL‐2: B cell lymphoma‐2
BCL‐xL: B‐cell lymphoma‐extra large
CAF: cancer‐associated fibroblasts
CAT: cancer‐associated thrombosis
CLEC‐2: C‐type lectin receptor 2
CypD: cyclophilin D
DAMPs: damage‐associated molecular patterns
DDS: drug delivery system
DIC: disseminated intravascular coagulation
DTS: dense tubular system
DVT: deep vein thrombosis
FAP: fibroblast activating protein
FDA: Food and Drug Administration
G‐CSF: granulocyte colony stimulating factor
Mcl‐1: myeloid cell leukemia 1
MOMP: mitochondrial outer membrane permeabilization
MPTP: mitochondrial permeability transition pore
NET: neutrophil extracellular traps
PAI‐1: plasminogen activator inhibitor‐1
PE: pulmonary embolism
PS: phosphatidylserine
Q‐VD‐OPh: Quinoline‐Val‐Asp‐Difluorophenoxymethylketone
RGD: Arg‐Asp‐Gly
ROS: reactive oxygen species
TF: tissue factor
TfR1: transferrin receptor 1
TLRs: toll‐like receptors
TNFR: tumor necrosis factor receptor
VDAC: voltage‐dependent anion channel
VEGF: vascular endothelial growth factor
VTE: venous thromboembolism

## 1 Introduction

Cancerous cells are mechanistically distinguished by their ability to successfully evade recognition by host immune surveillance, which triggers their eradication through the ignition of apoptosis [1, 2, 3]. This is a programmed demise mechanism in which the delicate balance between antiapoptotic and proapoptotic BCL‐2 (B cell lymphoma‐2) family proteins determines the fate of cells, with cancerous cells mainly improving their survival by disrupting this equilibrium [4, 5]. In this context, the alteration of the antiapoptotic BCL‐2 protein family, as a key regulator of apoptosis, is a main route through which different types of malignancies escape their eventual fate [6]. Hence, antagonizing these antiapoptotic proteins is considered a reasonable approach to prevent cancer progression, where apoptosis inducers such as BH3‐mimetics act as effective anticancer drugs through specific binding to the hydrophobic groove of BCL‐2 antiapoptotic proteins [7]. This is an interaction that causes proapoptotic proteins BAK and BAX to be released from their binding sites, oligomerize, and ultimately induce mitochondrial outer membrane permeabilization (MOMP), which triggers the apoptosis process [8].

However, as an important side effect, BH3‐mimetics usually decrease platelet counts, which may put patients at risk of thrombocytopenia [9]. This is due to the fact that, being annulated, platelets are much more vulnerable to apoptotic signals compared to other cells, which may have the capability of self‐restoration. In addition, contrary to other cells, platelet survival is mainly related to the abundance of BCL‐xL [10, 11] which can be effectively affected by BH3‐mimetics. In this way, the pharmacological inhibition of BCL‐xL leads to a significant decrease in platelet count, making the patients more vulnerable to hemorrhagic events [12]. Moreover, certain BH3‐mimetics may also disrupt calcium hemostasis by inducing an immediate calcium release, which depletes platelet stores and thus impedes the subsequent response to agonists [13] by presenting an exhausted phenotype of platelets. However, despite being insufficiently responsive to stimuli, these platelets are in turn procoagulant, which may cause an unwanted hypercoagulable state. This is an intricate situation in which some BH3‐mimetics may act as a “double‐edged sword” in inducing both thrombocytopenia and thrombotic events.

Accordingly, given the fact that patients with active cancer are at risk of both thrombotic and hemorrhagic events [14], an anticancer treatment with the least impact on platelet function and the coagulation system that does not exacerbate thrombophilic/hemorrhagic conditions would be a more favorable therapeutic choice. For this purpose, attention has shifted toward the new generations of BH3‐mimetics that have less effect on platelets by significantly reducing their affinity for BCL‐xL [15]. New‐generation BH3‐mimetics, such as venetoclax (ABT‐199), offer high selectivity for BCL‐2 and substantially reduce the risk of thrombocytopenia compared to earlier multitarget agents like navitoclax, which also inhibits BCL‐XL and destabilizes platelet survival [15, 16]. Nevertheless, it should be noted that thrombocytopenia still remains a common complication even in the latest versions of BH3‐mimetics. In this regard, while so far various types of BH3‐mimetics have been introduced to treat different cancers, mostly hematologic malignancies, including leukemia and lymphoma [17, 18], among these agents just one drug (venetoclax) successfully passed all preclinical and clinical phases to receive FDA approval [19]. To address this problem, apart from increasing the specificity of BH3‐mimetics, a targeted drug delivery system (DDS) also appears to be a practical approach that not only confers safe and efficient delivery of BH3‐mimetics to the cancer target but also prevents their nonspecific interactions with other cells and tissues, which may eliminate their adverse effects.

Therefore, to highlight aberrant side effects caused by BH3‐mimetics, the critical review presented here first introduces the mechanism behind their intervention in cellular necrotic and apoptotic pathways, particularly in platelets. Then further on, by introducing different versions of these drugs, the ongoing progress in the design of safer BH3‐mimetics with the highest effectiveness against cancer will be discussed. Finally, this review focuses on the development of anticancer drug delivery approaches to propose the most efficient platform for BH3‐mimetics to target cancer‐associated thrombosis (CAT). A platform that can target activated platelets and tumor cells with a single shot by loading a conventional BH3 mimetic onto a functionalized nanoparticle.

## 2 BH3‐Mimetics: Efficient Anticancer Drugs With Some Unwilling Effects on Platelets

The chemotherapeutic agents, BH3‐mimetics, have been developed to prevent cancer progression through interrupting the typical interaction between prosurvival and proapoptotic proteins within the BCL‐2 family [20]. Chemotherapy‐resistant neoplastic cells often prolong their lifespan mainly by suppressing the operative arm of mitochondrial‐driven apoptosis through upregulating antiapoptotic proteins belonging to the BCL‐2 family (BCL‐2, BCL‐xL, or MCL‐1) [21]. Based on this specific scenario, BH3 mimetics have so far shown promising results by neutralizing these antiapoptotic proteins and inducing cell death through activation of the intrinsic apoptotic pathway [22]. However, BH3‐mimetics have also been shown to interfere with other pathways of cellular demise including necrosis and necroptosis, which, in addition to apoptosis, may affect platelets and induce unwanted thrombocytopenia or thrombotic events during anticancer therapy [23]. Therefore, in the following sections, different cell death pathways that affect the function and survival of platelets with the intervention of BH3‐mimetics will be further discussed.

## 3 BH3‐Mimetics Effects on Different Pathways of Cellular Damage

**FIGURE 1:**
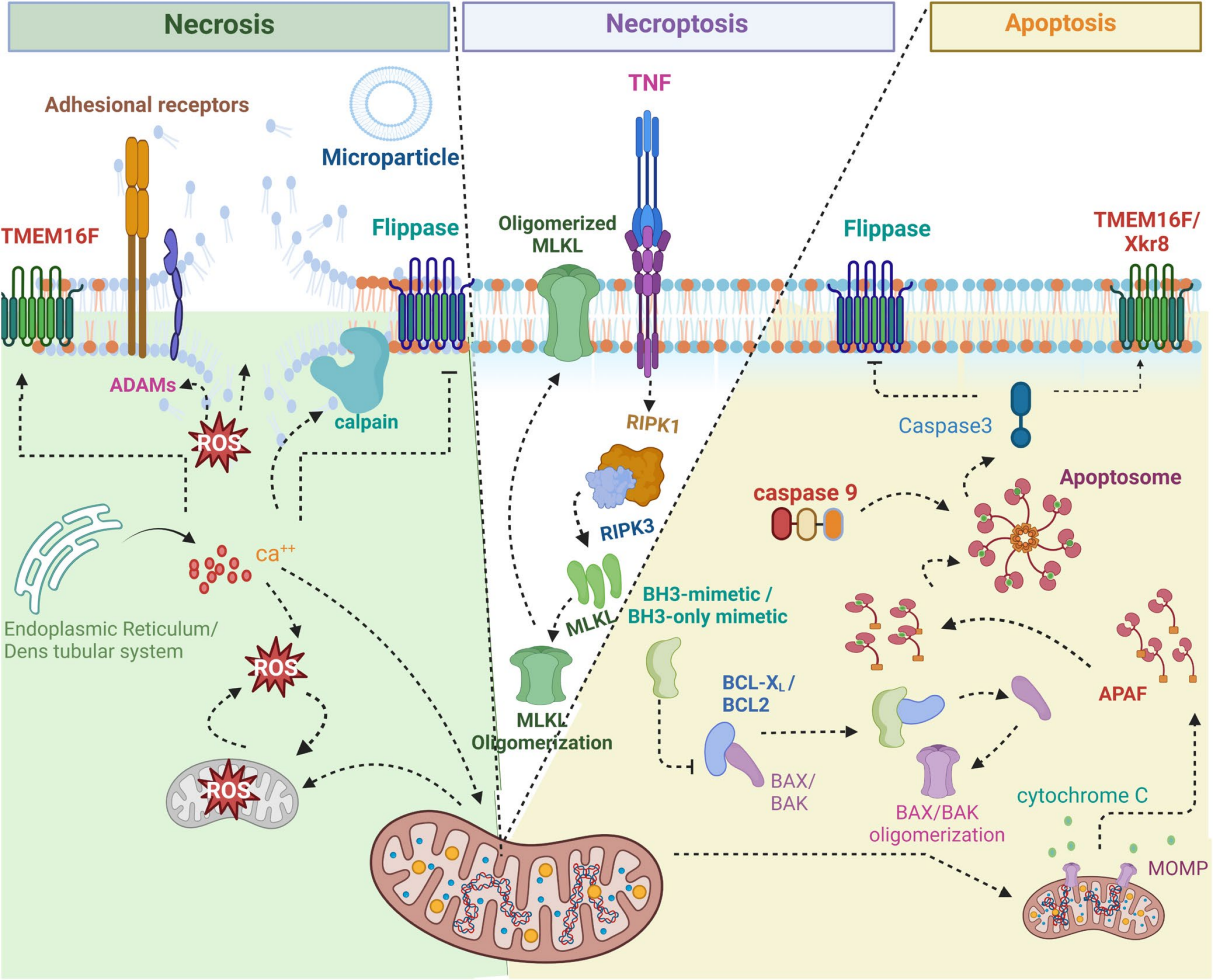
Different pathways of platelet demise. *Necrosis*: This pathway is orchestrated by Ca^2+^ influx, which triggers calpain activation, ROS generation as well as TMEM activation versus flippase inhibition, which leads to phosphatidylserine (PS) externalization and microvesiculation from the platelet membrane. Exaggerated cytoplasmic ROS accompanied by calcium influx synergistically triggers the formation of the mitochondrial permeability transition pore (MPTP), leading to the decline of mitochondrial membrane potential (Δψm) and ultimate depletion of ATP. Subsequently, elevated ROS can induce ADAMs activation, triggering the shedding of adhesion receptors where ROS generation also interferes with cellular membrane integrity. *Apoptosis*: BH3‐mimetics or BH3‐only mimetics block the prosurvival BCL‐2 family members such as BCL‐2 and BCL‐xL, resulting in the BAX and BAK oligomerization and MOMP formation on the outer membrane of mitochondria. The MOMP allows the release of cytochrome c, which then binds to APAF. Subsequently, caspase 9 joins the cytochrome c‐APAF complex, forming the apoptosome. The apoptosome, in turn, activates caspase 3, which once activated continues the apoptotic process while inhibiting flippase and promoting TMEM16F/Xkr8 activation, leading to externalization of PS. *Necroptosis*: The interaction between TNF and TNFR can trigger necroptosis by activating RIPK1 and RIPK3. Following the activation of RIPK1 and RIPK3, MLKL undergoes oligomerization and translocates to the cell membrane. This translocation leads to the cell membrane rupture and disruption of ion balance. ADAM, A disintegrin and metalloproteinase; APAF, Apoptotic protease activating factor; MLKL, Mixed Lineage Kinase Domain‐Like Pseudokinase; MOMP, Mitochondrial outer membrane permeabilization; MPTP, Mitochondrial permeability transition pore; RIPK3, Receptor‐interacting serine/threonine‐protein kinase 3; ROS, Reactive oxygen species; TMEM16F, Transmembrane protein 16 F; TNF, Tumor necrosis factor; TNFR, Tumor necrosis factor receptor; Xkr8, XK‐related protein 8.

Several lines of evidence have shown that different generations and versions of BH3‐mimetics can interfere with the main pathways of cellular demise, including necrosis, apoptosis, and necroptosis. Figure 1 summarizes the mechanisms behind these pathways.

## 4 The BH3 Mimetic Selective Recognition of Neoplastic Cells as a Challenge in Cancer Treatment

**FIGURE 2:**
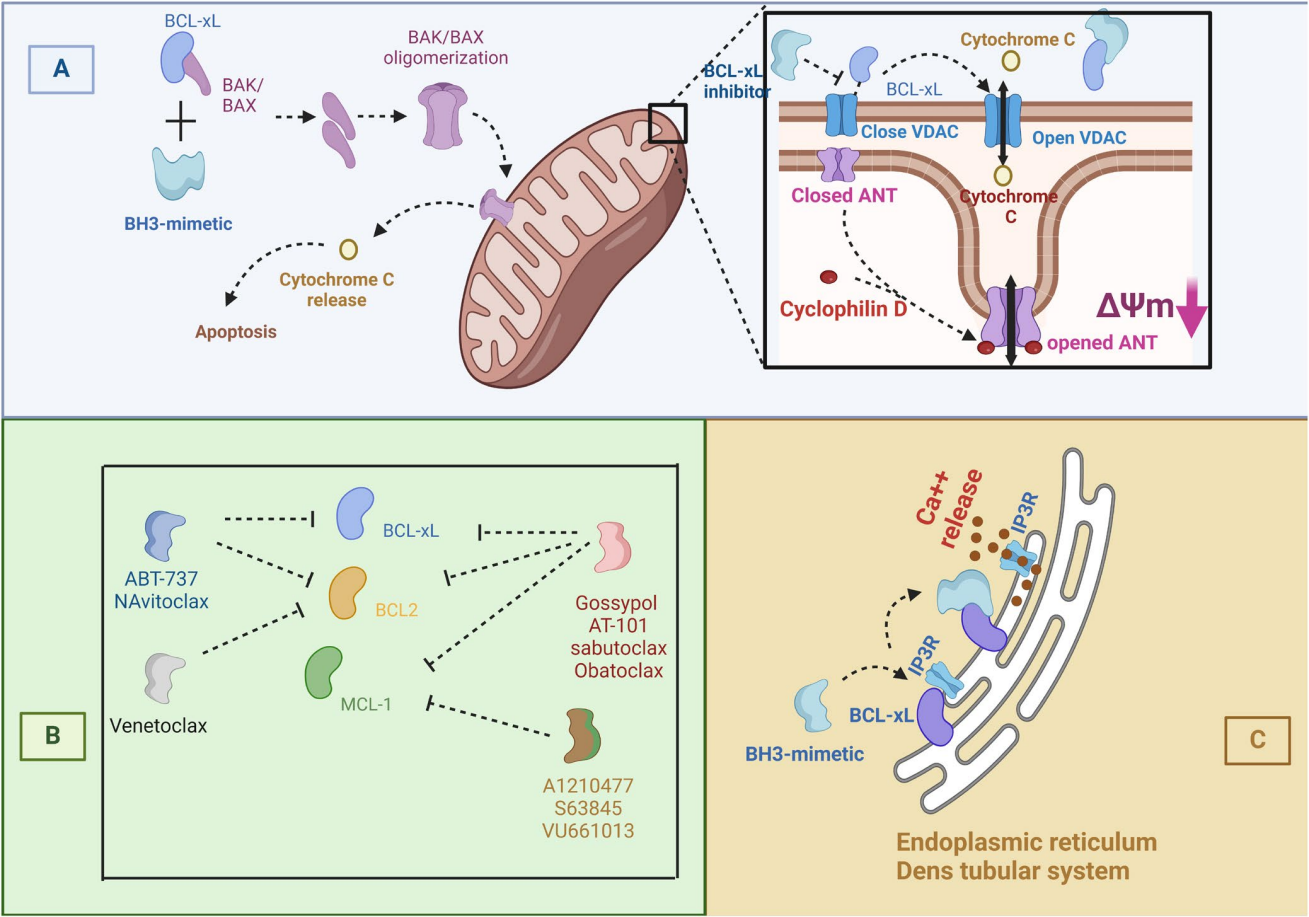
The interference of BH3‐mimetics with apoptotic and necrotic pathways. (A) BH3‐mimetics interfere with regular interaction between pro‐ and antiapoptotic BCL‐2 family proteins (BCL‐xL and BAK/BAX). This interference causes BAK/BAX oligomerization and induces MOMP formation, consequently triggering the apoptotic pathway through cytochrome c release. Furthermore, as depicted in the magnified image, antagonizing BCL‐xL by BH3‐mimetics may result in VDAC transition to open mode, resulting in the opening of MPTP and subsequent cytochrome c release, which further progresses cellular demise. (B) Gossypol, AT‐101, sabutoclax, and obatoclax act as pan‐BCL‐2 inhibitors suppressing all three prosurvival proteins of the BCL‐2 family, including BCL‐xL, BCL2, and MCL‐1. However, ABT‐737 and navitoclax cannot neutralize MCL‐1, whereas A1210477, S63845, and VU661013 only interact with MCL‐1, and in a similar way, venetoclax only binds to BCL‐2. As BCL‐2 proteins are also expressed on the endoplasmic reticulum, they play a critical role in Ca^2+^ hemostasis, where inhibition of these proteins by BH3‐mimetics induces Ca^2+^ influx (C). ANT, adenine nucleotide translocase, IP3R, inositol 3 phosphate receptor, MCL‐1, myeloid leukemia cell differentiation protein, MPTP, mitochondrial permeability transition pore, VDAC, voltage‐dependent anion channel.

The preliminary versions of BH3‐mimetics simultaneously antagonize multiple BCL‐2 antiapoptotic proteins, which caused “off‐target” apoptosis in both normal and tumoral cells [24, 25] (Figure 2A). However, later generations of BH3‐mimetics show stronger specificity that increases drug selectivity for cancer cells, thereby reducing the side effects of earlier classes [26, 27]. In this regard, it will be important to find a more distinct mechanism by which neoplastic cells can increase their survival and long‐term proliferation among normal cells, as they can be selectively targeted by this means. For example, some cancerous cells in lymphocytic leukemia/lymphoma such as chronic lymphocytic leukemia upregulate BCL‐2 protein to escape apoptosis [28, 29]. Therefore, for such cases, a specific BCL‐2 inhibitor could be a rational choice to selectively target these tumor cells [29]. Additionally, some leukemic cells develop their resistance to apoptosis by upregulating MCL‐1 expression [30], where a specific MCL‐1 inhibitor may be a logical selection for their eradication (Figure 2B). Hence, by preventing off‐target reactions, the application of more specific BH3‐mimetics could potentially overcome the side effects of these drugs against normal cells. Regardless of the low “on‐target” specificity of BH3‐mimetics, their interaction with different cellular pathways may also be involved in various clinical manifestations. In this regard, it has been shown that the aberrant interference of some BH3‐mimetics with necrotic or necroptotic pathways [23, 31] in addition to apoptosis may present new clinical evidence that adds more complexity to cancer treatment. An example of this situation is the simultaneous stimulation of both apoptotic and necrotic pathways of platelets by some BH3 mimetics, which can provoke thrombotic complications such as thromboembolism despite the occurrence of conventional evidence of thrombocytopenia [31].

## 5 BH3‐Mimetics, a Double‐Edged Sword of Thrombocytopenia and Thrombotic Events

Under typical circumstances, platelets undergo a hierarchical process throughout their lifespan, ultimately experiencing both necrosis and apoptosis [32]. However, certain agents such as BH3‐mimetics have the ability to bypass this process mainly via direct activation of apoptosis. As a consequence, thrombocytopenia is a common adverse effect of BH3‐mimetics, resulting from the antagonism of BCL‐xL, the primary platelet prosurvival protein belonging to the BCL‐2 family, which serves to impede apoptosis in a BAK/BAX‐dependent manner [33]. Since platelets are anucleated cells with a limited number of mitochondria, they are more susceptible to apoptosis when exposed to BH3 mimics than nucleated cells [34]. On the other hand, some BH3‐mimetics not only induce thrombocytopenia but also reduce platelet adhesive function due to the shedding of key platelet adhesion receptors GPIbα and GPVI [33], which, together with the induced procoagulant state, may increase the risk of thromboembolic events [31].

Alternatively, some certain BH3‐mimetics also induce platelet activation, which, together with increased exposure of PS, may indicate a necrotic phenotype of procoagulant platelets, supporting an increased risk of thrombosis and coagulopathy in vivo [31, 33]. Some studies have shown that since BCL‐xL is involved in the regulation of calcium release from the ER (endoplasmic reticulum) or possibly dense tubular system (DTS) in platelets through its interaction with IP3R and RYR (ryanodine receptors), the inhibition of this antiapoptotic protein has the potential to disrupt calcium homeostasis (Figure 2C) [35, 36]. On the other hand, BCL‐xL plays an important role in the regulation of calcium uptake by mitochondria with its effect on closing the Voltage‐dependent anion channel (VDAC), where the closure form of VDAC prevents cytochrome c and calcium release from mitochondria (Figure 2A) [37]. Therefore, the inhibition of BCL‐xL by BH3 mimetics may also result in mitochondrial‐dependent necrotic events, mainly due to significant release of calcium with the formation of MPTP [38]. In this regard, some studies have demonstrated that a specific group of BH3 mimetics, which antagonize BCL‐xL, induces immediate platelet calcium release and platelet activation upon administration, which may worsen the risk of coagulopathy by interfering with the normal function of platelets [33, 39].

## 6 Introducing Different Types of BH3‐Mimetics and Their Adverse Effects on Platelet Function

In this section, while introducing different versions of BH3 mimics (Table 1), their effects on proadhesive, proaggregatory, pro‐inflammatory, and procoagulant functions of platelets are discussed by reviewing their relevant preclinical studies.

**TABLE 1:** BH3‐mimetics and inhibitor therapeutic agents for malignancy.

BH3‐mimetics	Origin	Target	Developer	Types	Effect on platelet	Indication	Advanced to clinical phase	References
ABT‐737	Structure‐based design	Bcl‐2, Bcl‐xL	Abbott Labs. (IL, USA)	BH3‐mimetic	Induce apoptosis/disrupt calcium homeostasis/PS externalization	AML‐breast cancer‐ liver cancer	No	[40, 41]
Navitoclax (ABT‐263)	ABT‐737	Bcl‐2, Bcl‐xL, Mcl1	Abbott Labs. (IL, USA)	BH3‐mimetic	Induce apoptosis/disrupt calcium homeostasis/PS externalization	AML‐ALL‐lymphoma	Yes	[42]
Venetoclax (ABT‐199)	Navitoclax	BCL‐2	Abbott Labs. (IL, USA)	BH3‐mimetic	Slightly Induce platelet apoptosis	AML‐ALL‐MM	Yes	[43]
Gossypol AT‐101	Catton seed	Bcl‐2, Bcl‐xL, Mcl1		BH3‐mimetic	Induce nonapoptotic death‐ PS externalization	CML‐colon and prostat cancer—	Yes	[44]
Sabutoclax (BI‐97C1)	Gossypol	Bcl‐2, Bcl‐xL, Mcl1		BH3‐mimetic	Induce nonapoptotic death‐ PS externalization	Prostate, lung cancer	No	[45, 46]
TW‐37	Gossypol	BCL‐2, MCL‐1	University of Michigan (MI, US)	BH3‐mimetic	Slightly induce apoptosis‐ slightly PS externalization	Breast, prostate cancer	No	[47]
Obatoclax	In silico docking studies	Bcl‐2, Bcl‐xL, Mcl1	University of Montreal (CAN)	BH3‐mimetic	Induce thrombocytopenia	AML‐colon‐ Pancreas cancer	Yes	[48]
A1210477	High throughput screen	MCL‐1	AbbieVie (IL, USA) Genetech (CA, US)	MCL‐1 inhibitor	N/A	AML	No	[49]
S63845	In silico modeling	MCL‐1	Institut de Recherches Servier Oncology (FRA)	MCL‐1 inhibitor	N/A	AML, MM	Yes	[50]
VU661013	Structure‐based design	MCL‐1	Vanderbilt University (TN, USA)	MCL‐1 inhibitor	N/A	AML	Yes	[51]

Abbreviations: ALL, acute lymphocytic leukemia; AML, acute myeloid leukemia; CML, chronic myeloid leukemia; MM, multiple myeloma.

### 6.1 Nonspecific BH3‐Mimetics

#### 6.1.1 HA14‐1

HA14‐1 was one of the earliest versions of BH3‐mimetics that exhibit off‐target effects on normal cells. In this regard, some evidence indicates that HA14‐1 not only causes thrombocytopenia, but also activates platelets and leads to progression toward their necrotic phenotypes. These findings were confirmed when it was found that the activation of platelets through HA14‐1 is strongly dependent on the induction of calpain activity [31]. Consequently, for this reason, HA14‐1 was not considered appropriate for use in clinical trials.

#### 6.1.2 ABT‐737 and Navitoclax (ABT‐263)

ABT‐737 and navitoclax represent relatively safe and tolerable BH3‐mimetics that specifically target BCL‐2 and BCL‐xL, while not affecting MCL‐1 [52]. However, these compounds not only induce dose‐dependent thrombocytopenia but also trigger the shedding of the platelet main adhesion receptors, leading to a reduction in the adhesive function of platelets [53]. ABT‐737 and navitoclax also inhibit the function of GPIIb/IIIa, which potentially affects the normal aggregatory function of platelets [33]. However, the lack of interference of ABT‐737 with the pro‐inflammatory potential of platelets can be considered a positive aspect of this drug [54, 55]. The other limitation of ABT‐737 was its lack of oral bioavailability, which has been resolved by introducing navitoclax. That's why, with ABT‐737 overshadowed, the new drug navitoclax was selected for clinical trials instead. Nevertheless, the main obstacles hindering its progress to the clinical phase remained its biocompatibility and dose‐dependent thrombocytopenia [56, 57].

#### 6.1.3 Gossypol

Gossypol and its derivatives encompass a wide range of BH3‐mimetics that are derived from cotton seed extracts. These compounds have been identified through the use of NMR binding assays and fluorescence polarization displacement analysis [58, 59]. This particular subset of BH3‐mimetics is characterized as pan BCL‐2 inhibitors and exhibits behavior similar to NOXA‐like BH3‐mimetics [22]. Mechanistically, gossypol displays a specific interaction with BCL‐xL and has the potential to counteract the antiapoptotic properties of Bcl‐2 and MCL‐1. Furthermore, gossypol depletes cellular ATP through uncoupling oxidative phosphorylation, thereby triggering the generation of mitochondrial reactive oxygen species (ROS) [60, 61]. The occurrence of these events, concomitant with calcium release, may potentially induce necrosis in platelets, leading to a procoagulant‐like phenotype, mainly through nonapoptotic pathways [31]. This is confirmed by a recent study demonstrating that, unlike navitoclax and ABT‐737, the caspase inhibitor Quinoline‐Val‐Asp‐Difluorophenoxymethylketone did not effectively prevent PS externalization in response to gossypol‐derived compounds (AT‐101 and sabutoclax) [31]. Gossypol and its R‐(−) enantiomer, AT‐101, have been used as monotherapeutic agents or in combination with conventional chemo‐radiation in clinical phases. However, several studies have indicated that the clinical utilization of gossypol and AT‐101 is associated with coagulopathic events or cardiotoxicity, as evidenced by elevated levels of troponin [62, 63, 64, 65, 66, 67].

#### 6.1.4 Obatoclax

Obatoclax, also known as GX15‐070, is a pan BCL‐2 inhibitor that not only stimulates the intrinsic pathway of apoptosis but also initiates the activation of the extrinsic pathway via TRAIL‐mediated apoptosis [68, 69]. Furthermore, several lines of evidence indicate that obatoclax also triggers necroptosis [70]. In addition to these findings, some evidence also suggests that the clinical administration of obatoclax leads to platelet activation alongside thrombocytopenia. In this context, Chiappori et al. have demonstrated that obatoclax administration may not only cause thrombocytopenia and put patients at risk for hemorrhagic events, but it can also lead to deep vein thrombosis (DVT) [71]. A subsequent study conducted by Schimmer et al. has identified key indicators of thrombosis, comprising atrial fibrillation, acute MI, and cardiac murmur, which confirm the findings of Chiappori et al. [72]. Overall, despite several studies indicating the safety and tolerability of obatoclax, its administration is still associated with the occurrence of thrombosis and thrombocytopenia, which may pose a significant challenge in the treatment of cancer patients.

### 6.2 Specific BH3‐Mimetics

While nonspecific BH3‐mimetics show a wide range of inhibitory effects on proapoptotic BCL‐2 proteins, the next generation of BH3‐specific mimetics has been designed to significantly reduce the clinical complications of these drugs by eliminating their off‐target effects.

#### 6.2.1 Specific BCL‐xL Inhibitors

Since some cancer cells strategically enhance their survival by improving the expression of BCL‐xL, new versions of BH3‐mimetics such as A‐1155463 and A‐1331852 have been developed to specifically target BCL‐xL [73, 74]. However, none of these candidates have yet been approved for advancement to clinical trials as they induce thrombocytopenia in a manner similar to navitoclax.

#### 6.2.2 Specific MCL‐1 Inhibitors

The upregulation of MCL‐1 has been observed in a wide range of tumor cells and is linked to chemotherapeutic resistance in a broad range of cancers [75, 76]. There is evidence indicating that an elevated level of MCL‐1 is associated with tumor resistance to venetoclax and navitoclax [49, 77, 78]. MCL‐1 suppression alone has no inhibitory effect on the development of platelets and megakaryocytes [79]. However, when MCL‐1 inhibition is accompanied by BCL‐xL inhibitors, it synergistically develops the thrombocytopenic effect of BCL‐xL inhibitors [79]. It seems that these medications, when utilized as monotherapy, do not produce a substantial effect on platelets [79].

#### 6.2.3 Tw‐37

TW‐37, a second‐generation benzenesulfonyl derivative of gossypol, is a specific inhibitor of MCL‐1 and BCL‐2. Unlike the previous version of gossypol, TW‐37 has a minimal effect on platelets, which may be attributed to a significant decrease in its affinity for BCL‐xL [31]. Thus, while this drug retains its antineoplastic properties, it has reduced the usual complications associated with gossypol and its derivatives, with its lower effects on platelet activation and apoptosis [31, 80]. However, despite demonstrating robust antitumoral efficacy in preclinical investigations, TW‐37 has not yet advanced to the stage of clinical trials.

#### 6.2.4 Venetoclax (ABT‐199)

Venetoclax is a BCL‐2 inhibitor that is highly specific in its action and has been found to have minimal impact on platelets. Wei H et al. demonstrated that venetoclax does not induce PS externalization on platelets at a concentration of 10 μM [31]. Moreover, venetoclax does not demonstrate a significant impact on platelet mitochondrial impairment and disruption of calcium hemostasis [31]. However, several studies have reported that venetoclax induces thrombocytopenia during clinical phases [81, 82, 83, 84]. Nonetheless, there is no evidence to suggest that venetoclax induces platelet activation or thrombus formation. Furthermore, recently a piece of evidence indicated that some mutant variants of BCL‐2 in chronic lymphocytic leukemia (CLL) demonstrate resistance to venetoclax [85]. However, regardless of all these considerations, so far, venetoclax is the only BH3 mimetic that has successfully completed clinical trials and received FDA approval [86]. Notably, S65487/VOB560 is also considered a new BCL‐2 specific inhibitor that has advanced to the clinical trial phase [87]. Finally, despite the fact that some of the side effects caused by the off‐target reactions of the original version of BH3‐mimetics against platelets are no longer seen in the new generation of these drugs, the occurrence of thrombocytopenia even in venetoclax, as an FDA approved BH3 mimetic, is still an unsolved problem that requires further attention.

## 7 An Overview of Current Experimental Approaches to Enhance the Efficacy of BH3‐Mimetics Antitumor Drugs

There are three types of cancer, including hematologic neoplasms, metastatic, and localized cancers [88]. Hematologic neoplasms are the most commonly exposed cancers in the circulation [89]. The metastatic type is the most important cancer that invades and activates the endothelium and also induces serious inflammatory conditions [88]. Given the exposure of these two types of malignancies to the circulating blood, direct targeting of tumor cells or surrounding vasculature by drug compounds will be a feasible approach. However, localized tissue cancers that neither directly invade the endothelium of major vessels nor invade the blood are the most complex types of cancer to be treated with DDSs. There are several studies that have investigated different designs of pharmaceutical models of BH3‐mimetics in the treatment of various cancers, the most important of which are the following.

### 7.1 Strategies for Safe Transportation of BH3‐Mimetics: Extending Shelf‐Life and Maintaining Reactivity With Less Toxicity

The short shelf life of BH3‐mimetics or their nonspecific binding to plasma proteins such as albumin can affect the effectiveness of these drugs [90]. This is an important concern that, in some cases, will be challenging to compensate for by increasing the drug dose due to the increased likelihood of cytotoxicity. However, pharmacokinetic studies have suggested various strategies to enhance the durability and effectiveness of anticancer drugs so far, including peptide stapling [91], applicable to BH3 peptide [92], albumin fusion [93, 94] (in contrast to the diminishing effect of blood albumin), and dendrimer‐drug conjugate platforms [95, 96], the latter of which is currently applicable to BH3‐mimetics. The dendrimer‐drug conjugate significantly prolongs the drug's presence in circulation when compared to the drug alone. Furthermore, under these circumstances, the drug is also released gradually from the dendrimer nanocarrier within the bloodstream. Dendrimers are nanostructures formed by sequentially attaching polymer layers, leading to the development of a spherical shell that surrounds a central core. Dendrimers possess adjustable physicochemical properties, making them suitable for utilization as a DDS [96]. However, it should be noted that the dendrimer may also exhibit dose‐dependent cardiovascular toxicity, with low solubility and a strong affinity for plasma proteins [95, 96]. Thus far, this platform has been used for the safe delivery of many drugs, including BH3‐mimetics. An example of this is a study on the safe transportation of AZD4320, a dual Bcl‐2/Bcl‐xL inhibitor. In this study, in order to obtain the least toxicity and the best efficacy, Feeney et al. applied the conjugation of AZD4320 with a fifth generation (G5) poly‐l‐lysine dendrimer nanocarrier to minimize the side effects of the drug and increase its shelf life in the bloodstream. Intriguingly, this approach also achieved targeted accumulation of the drug at solid tumor sites [95, 97, 98]. Regardless of the cargo design using different materials, encapsulation is a useful method that can also be employed for successful dual‐drug delivery with minimal side effects, in addition to extending the shelf life of drugs, including BH3‐mimetics. In this regard, Bala et al. conducted a study where they utilized this platform to administer MCL1 or BCL2 inhibitors to mice. The results showed a significant reduction in the required dosage of the drugs, without any observed side effects such as weight loss or hematological toxicity. On the other hand, when the two inhibitors were administered without encapsulation, it resulted in significant weight loss and hematological toxicity affecting platelets, erythrocytes, WBCs, and hemoglobin [99]. Therefore, the transport of multiple drugs, minimization of drug dose to ensure less toxicity, and increased shelf life are three important advantages of the encapsulation strategy that have been successfully applied in the preclinical model of BH3‐mimetics.

### 7.2 Directional Drug Delivery System (DDS) for Antitumor Drugs Including BH3‐Mimetics

As already mentioned, one of the important challenges in the design of BH3‐mimetics is the ongoing effort to increase their specificity and efficiency against tumor cells in such a way that they can ensure the least toxicity to nontarget tissues. However, today the use of targeted delivery systems has partially overcome the exclusive reliance on the use of highly specific and efficient BH3‐mimetics with a “conserved conveyance” of these drugs to the specific location of the tumor. These unique systems enable precise targeting of the intended site without being influenced by the tissue‐inflammatory conditions typically observed in patients with underlying noncancerous diseases. Below are three of the most interesting and effective techniques documented in research.

#### 7.2.1 Nanoparticle Decorated With Inflamed Endothelial Homing Peptides

**FIGURE 3:**
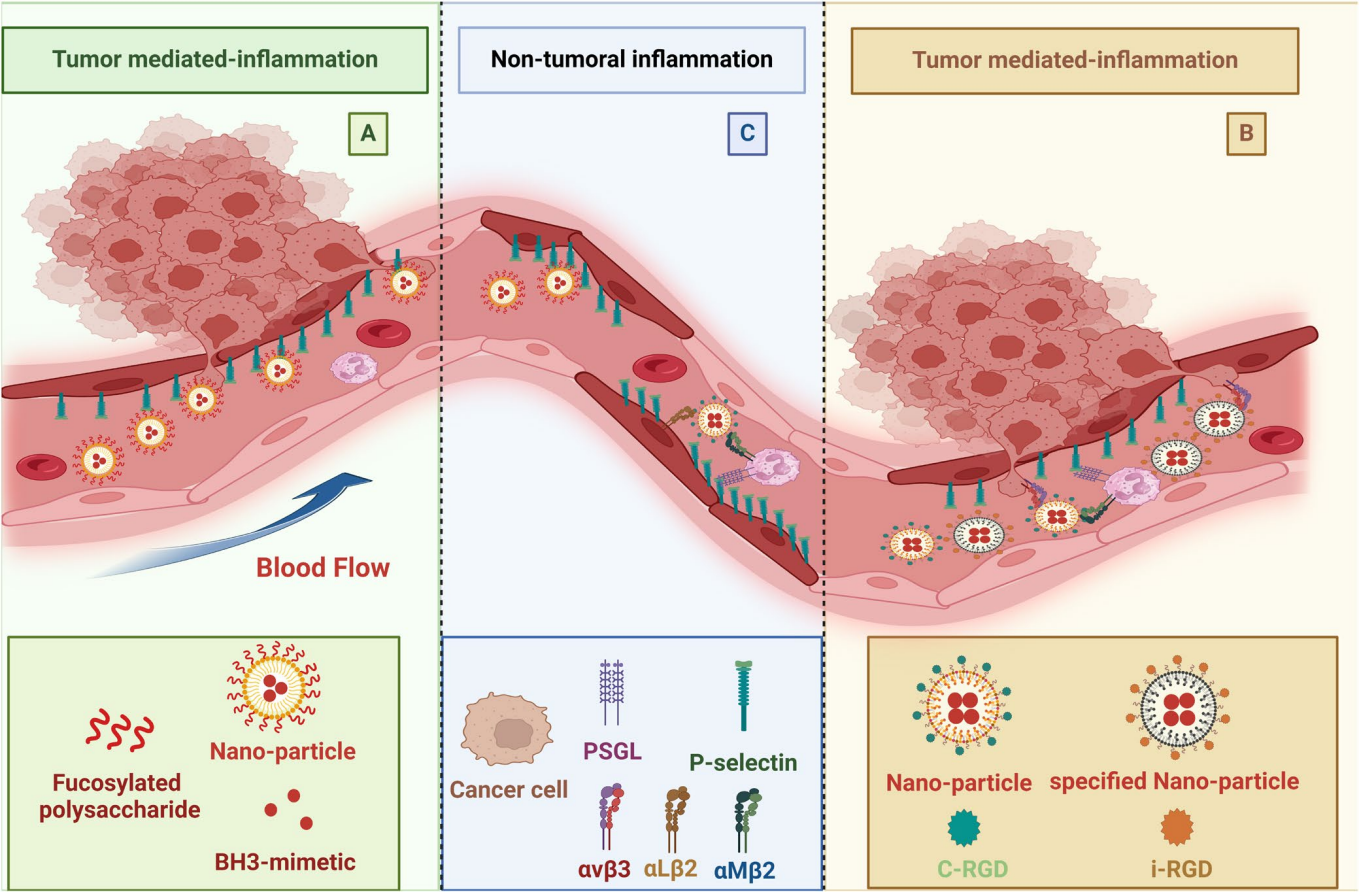
Directional drug delivery system (DDS) for BH3‐mimetics. (A) Nanoparticle decorated with inflamed endothelial homing peptides: Figure 3A shows cancer cell accumulation and metastasis through the endothelium, where the endothelial cells become activated and express inflammatory molecules including P‐selectin. A liposomal nanoparticle containing BH3‐mimetics (S63845 and venetoclax) is coated with fucosylated polysaccharide to selectively bind to P‐selectin. The nanoparticle adheres to the endothelial cells in close proximity to tumor vasculature. (B) Nanoparticle decorated with inflamed endothelial homing peptides: Figure 3B shows two different approaches to cancer targeting via liposomal nanoparticles decorated with RGD peptides as homing signals that detect and interact with integrins expressed by the tumor cells. Whereas BH3‐mimetics loaded nanoparticles with c‐RGD nonspecifically interact with several integrins, a unique RGD known as i‐RGD specifically targets αvβ_3_ integrin mainly expressed by tumor vasculature. (C) Nanoparticle decorated with inflamed endothelial homing peptides: Figure 3C highlights the nonspecific interactions of either fucosylated polysaccharide or c‐RGD decorated nanoparticles with nontumoral inflammatory milieu where the former reacts with P‐selectin expressed in inflamed endothelium, and the latter interacts with leukocytes and inflamed endothelial cells expressing integrins αMβ2 (MAC‐1) or αLβ2 (LFA‐1), respectively.

The design of the nanoparticle in this model considers a homing peptide in its structure to manage the delivery of the cargo to the closest vicinity of the tumor in a targeted manner, with the limitation that it is not specific to tumor cells per se but depends on tumor‐related conditions. When tumors invade the endothelium, their direct interaction can trigger the activation of the endothelial cells, while the ischemic conditions caused by the accumulation of tumor cells may also lead to endothelial dysfunction [100]. Consequently, for any of these reasons, endothelial cells located near the tumor express inflammatory markers that have been investigated in several animal studies [101, 102, 103]. Given this, Bala Thanan et al. have developed a directional delivery strategy for BH3‐mimetics (S63845 and venetoclax) that relies on targeting damaged endothelial cells in close proximity to the cancer. This strategy utilizes P‐selectin as a well‐expressed inflammatory receptor for convenient targeting of cancer‐associated endothelium. In this approach, the nanoparticle, carrying the fucosylated polysaccharide as a homing ligand for the P‐selectin receptor, possesses the ability to navigate precisely toward the damaged endothelium adjacent to the metastatic cancer, where, through membrane fusion, the nanoparticle transfers its contents into the cells (Figure 3A) [101].

However, it should be noted that such a design may not be biologically specific to the tumor and may interfere with other endothelial inflammatory conditions caused by different underlying diseases (Figure 3C), such as chronic obstructive lung disease [104], coronary heart disease [105], and ischemic cerebrovascular disease [106]. That is why other researchers are dedicated to finding a unique homing peptide for their drug carriers to specifically target the tumor in direct contact.

#### 7.2.2 Nanoparticle Decorated With Integrin Ligands Peptides

In a different model of nanoparticle design, Arg‐Asp‐Gly (RGD) motifs are utilized as the homing peptide. This design is mostly based on the high expression of α_v_β_3_ integrin, a well‐established primary receptor of RGD, on the surface of malignant vasculature. In tumor cells, α_v_β_3_ is highly expressed during tumor progression and induces cellular protrusion, regulation of cancer cell migration and invasion, and cellular contractility [107]. In addition, during the reaction with various ECM proteins, the association of this integrin with the receptors of vascular endothelial growth factor (VEGF) and FGF‐2 (fibroblast growth factor 2) can develop the invasiveness of cancer cells [108]. Studies have shown that utilizing RGD as a homing peptide offers several advantages, the most important of which is enhancing the endocytosis of drug carriers and thus improving drug uptake in the target tissue [109]. Furthermore, due to the upregulation of integrin α_v_β_3_ on the surface of tumor cells during cancer progression, RGD‐decorated nanoparticles show greater specificity to their pathological targets compared to other tissues and cells under resting conditions [109, 110]. In addition, laboratory studies have demonstrated that nanoparticles linked with RGD can effectively hinder the movement of tumor cells, which reduces the risk of metastasis [109, 110]. An example of this design is carried out by Loyer et al., in which they showed enhanced absorption of cyclic RGD (cRGD)‐labeled nanoparticles encapsulating drugs into hepatoma cells compared to nanoparticles lacking RGD [110]. However, regardless of labeling nanocarriers with RGD, the structural nature of the carrier itself is also important, which has been the subject of methodological studies.

The use of nanocarriers with a polysaccharide structure such as chitosan is one of the methods that have been applied for the successful design of targeted delivery of antitumor drugs [111]. It is worth mentioning that, in addition to being a suitable nanocarrier due to its biodegradability and biocompatibility, one of the most important advantages of CHNPs (chitosan nanoparticles) for drug delivery is their positive charge, which allows them to easily pass through the cell membrane and increases the ability of drug endocytosis [112, 113, 114, 115]. In this regard, Yadav et al. conducted a study in a breast cancer cell environment in vitro, which showed that raloxifene delivered by CHNPs containing RGD significantly inhibited cell migration and angiogenesis [111].

Liposome‐based designs are also considered as another drug delivery method with a wider scope of research, which also includes BH3‐mimetics [109]. One notable study in this field is the research conducted by Liu et al., where they focused on the directional delivery of BH3‐mimetics (Gossypol) by designing liposome‐based nanoparticles that were conjugated with RGD. The findings of this study demonstrated that liposomes conjugated with RGD exhibited excellent biocompatibility and had a significant inhibitory effect on PC‐3 (prostate cancer) in mice [109].

Today, the use of RGD as a potent tumor biomarker represents a major breakthrough in different cancer treatment approaches, including integrin‐targeted molecular imaging and nanotechnology‐driven drug treatments [116]. Nevertheless, the lack of selectivity of some conventional RGD peptides and their interaction with multiple integrin receptors may reduce the effectiveness of these approaches with nontargeted attachments, thus compromising their strength [107]. In addition, the specificity issue could result in more complex situations when engaging with other mechanisms, ultimately impeding the growth and spread of cancer cells, especially in cases of cross‐reactivity with the inflammatory milieu and immune cells [107]. On the other hand, due to cross‐binding of nonspecific RGD peptides to the integrins expressed by activated platelets and leukocytes, such drug carriers with no exclusive binding to tumor vasculature may be act as a “double‐edged sword” with antitumor benefits versus some possible adverse effects including hemorrhage or interference with the functional activities of leukocytes even against tumor progression [117, 118, 119]. This is why today many efforts have been devoted to the design of various RGD peptides that specifically target tumor‐associated integrins in order to enhance the effectiveness of anticancer medications.

One of the well‐established specific RGDs that has been able to successfully pass preclinical studies and move to clinical trial in the field (NCT03517176) is iRGD (internalizing RGD), a tumor‐penetrating disulfide bridge harboring peptide (cyclo[CRGDK/RGPD/EC]) which was identified to target αv integrins expressed on endothelial cells of tumor vessels including both αvβ3 and αvβ5 [120, 121, 122, 123, 124]. Following integrin binding, the proteolytic cleavage of iRGD within the tumor forms the peptide sequence CRGDK/R which interacts with NRP‐1, a membrane‐bound coreceptor of different ligands including VEGF. With this interaction, iRGD combined with nanocarriers penetrates deeply into extravascular tumor tissue [116].

In this regard, one of the promising preclinical studies for the treatment of breast cancer was conducted by Hu et al. where they introduced an in vivo model of 4 T1 breast cancer treatment with the coadministration of the tumor‐homing peptide iRGD and multistage‐responsive penetrating nanoparticles, consisting of a hyaluronic acid (NH) shell modified with a NO donor and a dendrimer linked to doxorubicin and indocyanine [125].

Thus far, given several preclinical studies and the abovementioned clinical trial which provided promising results in the treatment of different malignancies such as colon and colorectal cancer [126, 127], breast cancer [125], and metastatic pancreatic ductal adenocarcinoma [120], the incorporation of BH3 mimics into iRGD‐decorated nanocarriers also seems to be a viable strategy to increase drug efficacy with targeted delivery and minimal side effects, which may even eliminate the need for more specific versions of these drugs.

#### 7.2.3 Nanoparticle Decorated With Different Arrays of Homing Peptides With the Ability to Pass Blood Barriers While Penetrating Tumor Cells

**FIGURE 4:**
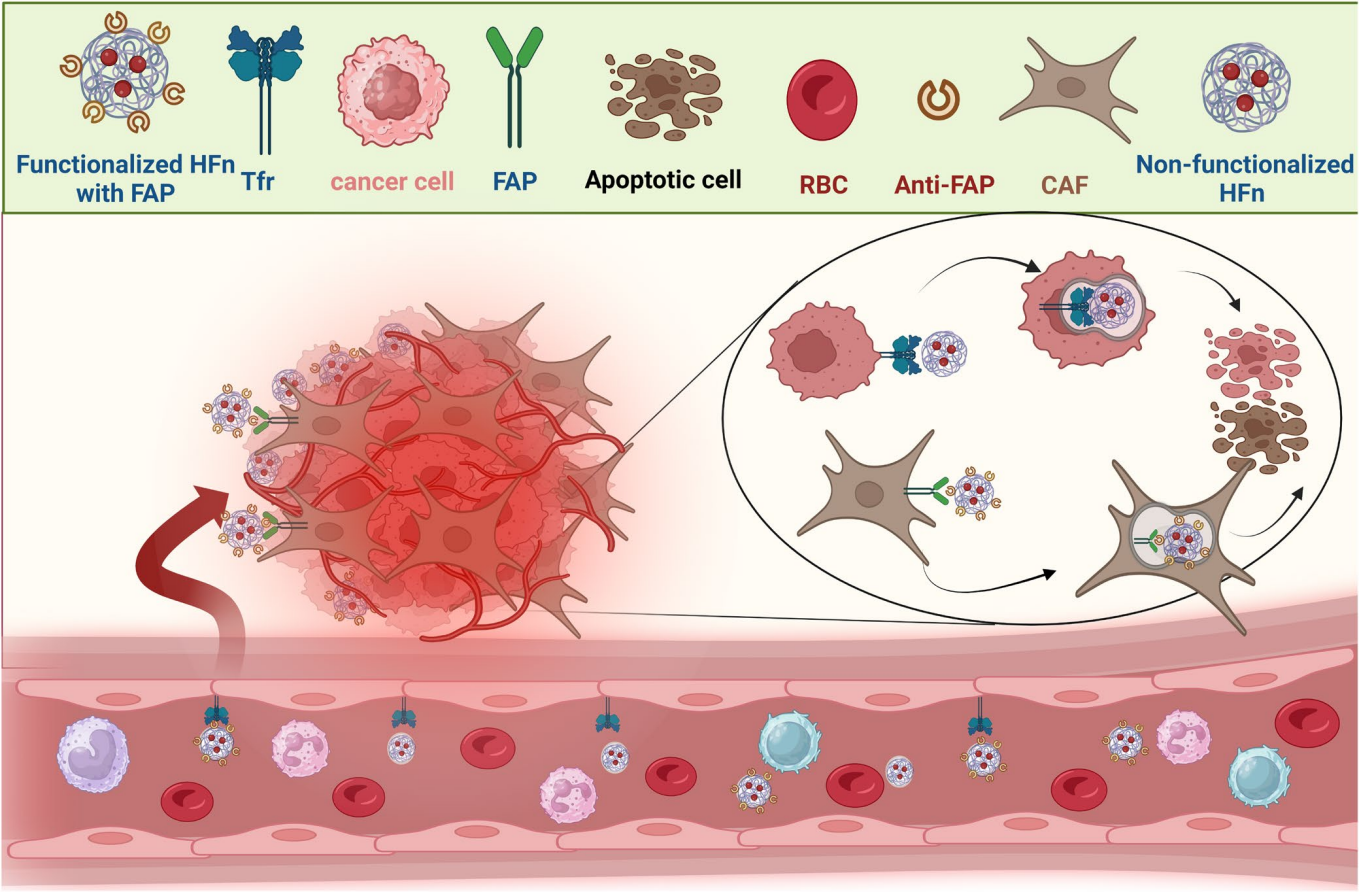
Targeting solid tumors through HFn nanocage loaded with BH3‐mimetics. The HFn nanocage, loaded with BH3‐mimetic either coated with or without anti‐FAP fragment, has the ability to cross blood vessels by interacting with endothelial TfR‐1 and directly targeting solid tumors. By functionalizing the HFn with the anti‐FAP fragment, it specifically detects CAF, initiating the process of apoptosis. However, the uncoated HFn (bare one), loaded with BH3‐mimetic, also recognizes tumor cells expressing TfR‐1. CAF, cancer‐associated fibroblasts, FAP, fibroblast activated protein, HFn, heavy chain ferritin, TfR‐1: transferrin receptor‐1.

Ferritin, a natural nanocarrier, serves as an excellent nanoparticle for effective drug transportation, enabling an accurate drug delivery to target cells and facilitating the transport of substantial amounts of therapeutic cargo [128]. Moreover, it demonstrates improved physicochemical properties and biocompatibility, making it an ideal choice for drug delivery [129]. This molecule consists of HFn (heavy chain ferritin) and LFn (light chain ferritin), wherein HFn exhibits superior quality for drug delivery. HFn possesses the capability to cross through the blood barrier to access extravascular tumors and directly recognize cancer cells via the interaction with transferrin receptor 1 (TfR1), which is expressed on both tumor and endothelial cells [130]. This interaction facilitates the rapid internalization of HFn nanoparticles into endothelial and tumor cells. Moreover, the ability of HFn to be functionalized with specific proteins [131] has led to the use of this nanoparticle in several studies to specifically target cancer (Figure 4). In this regard, Sitia et al. [132] employed HFn as a nanocage loaded with navitoclax, which was also functionalized by incorporating an anti‐FAP (fibroblast activating protein) antibody fragment to specifically recognize cancer‐associated fibroblasts (CAF) with high expression of FAP. Their findings indicated that the functionalized HFn substantially improved the specificity and cytotoxicity against solid tumors as compared to nonfunctionalized HFn. In another study, to directly target the cancer cells through α_v_β_3_ integrin, Shuai et al. employed an HFn nanocage functionalized with RGD motifs that was loaded with truncated BH3 domain. Their findings also revealed a greater induction of apoptosis in glioma cancer cells (C6 cell line) when treated with HFn‐BH3mimetic functionalized with RGD, as compared to bare HFn‐BH3mimetic or BH3mimetic alone [133]. Other studies also employed HFn for in vivo targeting of solid tumors, particularly glioma [130, 134], which have illustrated the successful transfer of HFn across the blood–brain barrier, enabling specific targeting of tissue cancer cells.

## 8 Cancer Associated Thrombosis (CAT): Evidence for Mutual Cross‐Talk Between Tumor Cells and Platelets

The cancerous state is usually associated with the disturbance in Virchow's triad, including blood flow stasis, endothelial damage, and hypercoagulability, which may lead to thrombotic complications due to improper function of the coagulation and fibrinolytic pathways as well as platelet activation [135, 136]. CAT is mostly manifested as venous thromboembolism (VTE), including DVT and pulmonary embolism (PE) [137]. However, there are also some studies that report arterial thrombosis in cancer patients [138, 139, 140] in the absence of an atherosclerotic plaque, where systemic hypercoagulation induced by factors secreted from cancer cells, such as thrombin and VEGF as well as chemotherapy adverse effects, can be associated with this thrombotic condition [140]. However, it should be noted that thrombotic events under a cancerous state are not just limited to these two classic forms of thrombosis, as other severe and rare thrombotic complications, including DIC and thrombotic microangiopathy [141], can occur in some patients, manifested by a consumptive coagulopathy resulting in microvascular thrombosis with a tendency for severe bleeding, thrombocytopenia, and organ failure [142, 143, 144, 145].

In general, CAT is caused by both direct and indirect interplay between tumors and platelets [135], where circulating or metastatic tumor cells stimulate platelet activation and coagulation [146], while, on the other hand, activated platelets also play an important role in cancer progression by increasing tumor growth, angiogenesis, and metastasis, which also promote cancer‐related thrombosis in a vicious circle [147]. Mechanistically, in a direct crosstalk, platelet recruitment by tumor cells can be facilitated by the interaction of platelet P‐selectin with glycoprotein s‐Le (x) on mucin‐producing carcinomas or sulfatides expressed on specific cancer cells, while the interactions also more strengthen with interplays between platelets GPIb‐IX‐V, GPIIb‐IIIa (α_IIb_β_3_), and tumor cell integrin α_v_β_3_ [148]. CAFs and pancreatic cancer cell lines have also been shown to express PDPN (podoplanin) [149, 150, 151], a protein known to induce platelet activation and aggregation through the CLEC‐2 (C‐type lectin receptor 2) [152]. In addition to tumor‐induced platelet activation, which may progress to a procoagulant state by platelets expressing PS, another key player in hypercoagulability is tumor‐associated expression of tissue factor (TF) [153], which, in complex with factor VIIa, plays a crucial role in blood coagulation, inflammation, and angiogenesis [154], further developing CAT. Moreover, TF is involved in the regulation of α_v_β_3_ integrin expressed on platelets, cancer cells, and endothelium [155], the main integrin whose activation contributes to tumor angiogenesis and progression [156]. On the other hand, the role of plasminogen activator inhibitor‐1 (PAI‐1) expressed by tumor cells is also of great importance, as the involvement of this fibrinolysis inhibitor has been shown to be correlated with an increased risk of thromboembolic events, especially in patients with pancreatic cancer [157, 158]. In such a milieu, the direct interplay between recruited neutrophils, inflamed endothelial cells, and tumor cells can also result in neutrophil extracellular traps (NET) formation, which adds further complexity to the preexisting hypercoagulative state [159].

Regardless of direct cellular cross‐talk, the continuous release of different agonists, including thrombin and ADP, by both tumor cells and developing thrombi, as well as tumor‐released cytokines, including TNF‐α and IL‐1β, enhance platelet activation and adhesion while impairing the antithrombotic responses of endothelial cells. Other biomaterials, such as DAMPS, VEGF, G‐CSF, and basic fibroblast growth factor, are also important mediators that contribute to the procoagulant phenotype of host cell [135].

## 9 Nano‐Drug Delivery Against Cancer Associated Thrombosis, Whether It Can Be a Therapeutic Choice

Similar to anticancer therapies, various designs have been introduced by different studies on the targeted delivery of antithrombotic drugs to the site of thrombotic events [160, 161, 162, 163]. In the same case scenario, given the overexpression of α_IIb_β_3_ integrin by activated platelets on developing thrombi [164, 165], drug conjugation or labeling drug‐holding nano‐carriers with RGD residues can also be considered a convenient approach to controlling thrombotic complications [166, 167, 168].

Perhaps one of the earlier studies of this kind in platelet biology is that of Gupta, Anirban Sen, et al., in which they used liposomes bearing surface‐conjugated linear RGD peptide to target and bind activated platelets [166]. Years later, cRGD was replaced in platelet nanomedicine as the most convenient homing peptide for the design of different nanoparticles used for antithrombotic drug delivery, of which the two studies presented by Zhang et al. and Huang et al. were classic examples, employing cRGD‐functionalized liposomes encapsulating urokinase and tPA‐loaded, cRGD‐coated, PEGylated liposomes as a nano‐carriers of a fibrinolytic drugs, respectively [167, 169]. Most recently, Xu et al. have also developed a biomimetic targeted thrombolysis approach through the construction of a RGD peptide‐modified RBC membrane‐functionalized nanoparticles, coloaded with urokinase and PFP (perfluoro‐n‐pentane). The approach aimed to achieve an effective nanomedicine with an acceptable hemo‐ and cytocompatibility [170]. However, although there have been several studies in this decade on fibrinolytic drugs loaded in RGD nanoparticles, no preclinical studies or clinical trials have been conducted on the loading of antiplatelet drugs using these nano‐carriers.

To avoid the possible side effects of nanomedicines, considerable efforts have been devoted to increasing the specificity of RGD peptides for differential targeting of integrins involved in various diseases. For example, while being a specific RGD mimetic, the antiplatelet drugs eptifibatide and tirofiban were designed to show the highest affinity for α_IIb_β_3_; however, they have the lowest capacity for interaction with α_v_β_3_ and other αv integrins [141, 171]. On the other hand, Cilengitide, a cyclic RGD pentapeptide with the highest specificity for integrin α_v_β_3_, α_v_β_5_, and α_5_β_1_ (IC50s of 0.61 nM [α_ν_β_3_], 8.4 nM [α_ν_β_5_] and 14.9 nM [α_5_β_1_], respectively) and the lowest affinity for α_IIb_β_3_ (IC50s of 5400 nM), can be a selective choice for cancer treatment with promising results that ensure the lowest risk of bleeding due to minimal interference with platelet integrin [172, 173].

However, it should be noted that in more advanced stages of cancer, especially in metastatic conditions, thrombotic complications and the possibility of thromboembolism are important risks that require special attention. It is necessary to provide antithrombotic treatments along with conventional antitumor therapies [135, 138, 140, 174]. Therefore, in such a situation, it may be useful to integrate anticancer and antithrombotic treatment strategies into a coherent design model of nano‐carriers.

To achieve this goal, focusing on specific RGD peptides to interact with β_3_ integrins may present a unique opportunity for the design of an effective dual therapies against cancer‐associated thrombotic events [119, 175]. This allows researchers to utilize nanoparticles decorated with RGD motifs that have a high affinity for the major integrins α_v_β_3_ and α_IIb_β_3_ simultaneously to effectively target both tumor cells and activated platelets involved in thrombotic complications, especially in a metastatic cancerous state [176, 177]. Whilst for this purpose, some RGD peptides such as Echistatin with the broad affinity pattern to α_IIb_β_3_, α_v_β_3_, and α_5_β_1_ might be suggested [173, 178, 179], other ligands like Syndecans, which have been shown to be involved in β_3_‐mediated extracellular vesicle capture [180], can act as a suitable homing peptide to direct nano‐carriers to the site of CAT. Other molecules such as heparan sulfate proteoglycans, whose interaction with β_3_ can lead to clathrin‐mediated and thus dynamin‐driven endocytosis, may also enhance drug penetration [181].

Considering the abovementioned strategy for simultaneous targeting of tumor cells and activated platelets by targeting β_3_ integrin, the design of a suitable nano‐carrier to deliver effective dual antithrombotic and anticancer drugs would be the next step to complete a DDS against tumor‐associated thrombosis. In this regard, although the strategy of using a common carrier to target β_3_ may seem justified in patients with a significant risk of thrombotic events in the background of invasive cancer, it appears that with some considerations, it can also extend to other patients, with less fear of unwanted hemorrhagic risk.

One important consideration is that both tumor cells and procoagulant platelets in more advanced stages of thrombosis can release a significant amount of thrombin, a proteolytic enzyme that, in addition to enhancing drug permeability, also reflects the further stage of platelet activation [165, 182, 183]. Here, it seems that targeting thrombin‐producing procoagulant platelets can be a good choice from the point of view that the occurrence of these platelets indicates a more advanced condition of thrombosis. This is a situation that, beyond the risk of possible bleeding due to the inhibitory effect of the antiplatelet drug, if not taken care of, can lead to the development of secondary thrombosis and the risk of thromboembolism in uncontrolled conditions. Especially with this consideration that procoagulant platelets have less adhesive properties due to the shedding and down‐regulation of platelet adhesive receptors [184, 185, 186].

With the above explanation, a particularly attractive nanoparticle design in this context is the one suggested by Absar et al., which includes RGD as a homing peptide and employs thrombin‐sensitive peptides to package the antithrombotic drug tPA within an albumin protection cage [187].

In general, the basis of this design is that after directing the drug nano‐carrier to the integrin‐expressing thrombus by RGD peptide, the breaking and opening of the drug‐holding cage takes place only in the presence of thrombin. Consequently, during CAT targeting, as thrombin is released either from the growing procoagulant thrombus or from invading tumor cells, the released drug primarily affects the more advanced stages of thrombus formation. Therefore, this design attenuates the risk of bleeding by reducing the possibility of inappropriate drug distribution and its interaction with immature thrombi.

However, in order to increase the specificity of drug delivery, instead of RGD against β_3_ integrins, which simultaneously detects activated platelets and tumor cells, iRGD can also be used for the exclusive targeting of tumor integrins. Although this design may have a lesser antithrombotic effect, it can still have significant efficacy due to the incorporation of thrombus into the tumor vasculature.

Now, taking all these considerations into account, it seems that by carrying a cargo containing dual antiplatelet and anticancer drugs in an RGD‐decorated nanoparticle, especially in a design similar to Abser et al., it would be possible to efficiently target CAT. In this regard, the fact that some nonspecific BH3‐mimetics can effectively exert both antitumor and antiplatelet effects [71, 84] suggests that their loading in targeted anti‐CAT nanocarriers could be a unique advantage due to their broad‐spectrum anticellular reactivity. One example of these BH3‐mimetics with promising antitumor effects is ABT‐737, which has also been experimentally shown to have prominent apoptotic effects on platelets [188] (Table 2). Although systemic administration of ABT‐737 was associated with adverse antiplatelet effects such as thrombocytopenia, it could be a suitable choice for dual targeting of tumor cells and platelets in a nanocarrier‐based treatment of CAT.

**TABLE 2:** Nano‐drug delivery strategies for targeted therapy of cancer‐associated thrombosis.

Nanocarrier core	Homing peptide	Primary target	Stimuli‐sensitive element	Drug encapsulated	Model	Effect on CAT	References
Liposomes	Linear RGD	Activated platelets (αIIbβ3)	None	None	In vitro	Selective binding of RGD‐liposomes to activated platelets, enabling integrin‐specific platelet targeting, a prerequisite for localized CAT intervention.	[166]
PEGylated liposomes	Cyclic RGD (cRGD)	Activated platelets (αIIbβ3)	None	tPA (tissue plasminogen activator)	In vivo	tPA‐loaded, cRGD‐coated liposomes preferentially localized to thrombotic sites, improving thrombus dissolution while minimizing off‐target bleeding risk in CAT.	[167]
Albumin cage + liposomes	Linear RGD	Activated platelets (αIIbβ3) and thrombin‐rich thrombi	Thrombin‐sensitive peptide	tPA	In vivo	Thrombin‐activated release of tPA from RGD‐guided nanocarriers selectively acted on advanced thrombi, reducing bleeding risk and improving safety in CAT treatment.	[187]
Liposomes	cRGD	Activated platelets (αIIbβ3)	None	Urokinase	In vivo	cRGD‐liposomes delivered urokinase directly to platelet‐rich thrombi, enhancing clot lysis efficiency and reducing systemic fibrinolysis‐related risks.	[169]
RBC membrane‐coated nanoparticles	Linear RGD	Activated platelets (αIIbβ3)	None	Urokinase + PFP (perfluoro‐n‐pentane)	In vivo	Biomimetic RGD‐nanoparticles coloaded with urokinase and PFP achieved efficient thrombus targeting and ultrasound‐activated fibrinolysis, improving hemo‐ and cytocompatibility in CAT models.	[170]

Abbreviation: RGD, Arg‐Asp‐Gly.

## 10 Conclusion

### 10.1 A Suitable and Feasible Design for Directional Targeting of CAT

**FIGURE 5:**
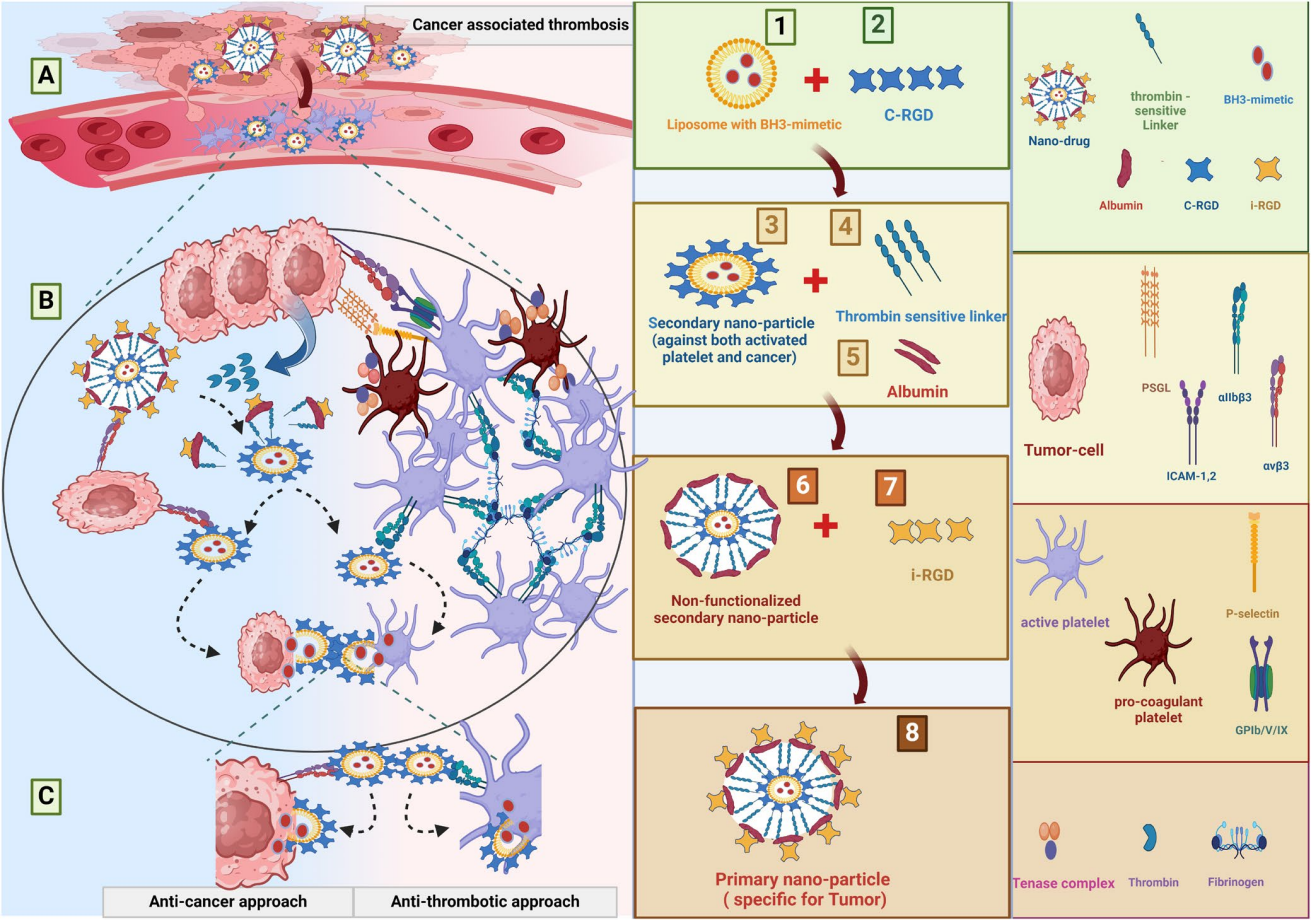
Targeting Cancer associated thrombosis (CAT). The left panel (A) shows cancer‐associated thrombosis with activated platelets expressing α_IIb_β_3_ in close contact with tumoral vasculature expressing α_v_β_3_ integrin, where both cells release a significant amount of thrombin. In the middle panel (1–8), the suggested design for RGD‐decorated nano‐particle has been described. Here, a secondary nano‐particle (3) consists of a liposomal core that is loaded with BH3‐mimetics (1) and coated with c‐RGD (2). The secondary nano‐particle is then enclosed with thrombin‐sensitive linkers (4) in an albumin cage (5), resulting in the formation of a primary nano‐particle (6) that is subsequently functionalized with i‐RGD (7), which converts it to the primary nano‐particle (8). As shown in the magnified image (B), the primary nano‐particle specifically recognizes the α_v_β_3_ integrin expressed by tumor cells, where, in the presence of thrombin, the thrombin‐sensitive linkers are cleaved, releasing the secondary nano‐particle into the metastatic cancerous site. The secondary nano‐particle can then recognize either activated platelets or tumor cells' integrins through their c‐RGD. Upon binding to the cancer or activated platelets, the BH3‐mimetics enter the cytoplasm of the tumor cells or activated platelets, triggering the apoptotic pathway (C).

Now, putting together all the available designs and considering the strengths and weaknesses of each, it appears that loading a BH3‐mimetic like ABT‐737 with a dual antitumor and antiplatelet effects in a liposomal carrier decorated with RGD peptides against integrin β_3_ or iRGD (specifically against α_v_β_3_) could be a practical design to limit or eliminate CAT. However, this model can be made more sophisticated with decoration by thrombin‐sensitive peptides linking the liposomal cargo to an albumin cage covered by a second layer of RGDs, here iRGD which specifically targets α_v_β_3_ mainly expressed by tumor vasculature. In such a design, while the external iRGD motif specifically directs whole particles to the site of cancer invasion, the proteolytic effects of thrombin and the release of the drug‐containing liposomal core decorated with the first layer of RGD against β_3_ integrin can react with both activated platelets and tumor cells in one shot. This RGD peptide also increases the drug penetration into the target cells by enhanced liposome incorporation with the cell membrane (Figure 5).

### 10.2 Future Perspectives

Given this potential model and with a view to translational approaches, it is suggested to focus on some key areas for future studies. First of all, appropriate in vivo experiments are needed to confirm the effective dual targeting of the designed nanocarrier model against cancer and thrombosis. If the ambient targeting is achieved, the spatial and effective release of BH3 mimetics (here ABT737) should be established such that the cytolytic effect of the drugs against both platelets and tumor cells is confirmed in the in vivo CAT model. To achieve this goal, the successful breakage of primary nanoparticles is essential, where cleavage of thrombin‐sensitive linkers in response to endogenous thrombin release from CAT must be optimized. Considering all the measurements performed, along with other models such as systemic administration of BH3 mimetics or similar DDSs, the optimal efficacy and effective targeted therapy of this new design should be confirmed and compared in preclinical and clinical studies in terms of minimal side effects, especially thrombocytopenia. Importantly, the main advantage of this targeted therapy is the minimal interference of the proposed DDS with natural hemostasis, in which the drug only attacks cancer‐related thrombosis, not other forms of physiological thrombus generation that ensure natural hemostasis. This is another important issue that should be carefully addressed in both in vivo models and preclinical studies.

## Author Contributions

**Mehran Ghasemzadeh:** conceptualization (lead), data curation (lead), funding acquisition (lead), investigation (lead), methodology (lead), project administration (lead), supervision (lead), validation (lead), visualization (lead), writing – original draft (lead), writing – review and editing (lead). **Nazanin Heidari:** conceptualization (supporting), data curation (supporting), investigation (supporting), writing – original draft (supporting). **Jalal Naghinezhad:** conceptualization (supporting), data curation (supporting), investigation (supporting), writing – original draft (supporting). **Alireza Ghasemzadeh:** conceptualization (supporting), data curation (supporting), investigation (supporting), methodology (supporting), writing – original draft (supporting). **Ehteramolsadat Hosseini:** conceptualization (equal), data curation (equal), investigation (equal), methodology (equal), supervision (equal), writing – original draft (equal).

## Ethics Statement

The authors have nothing to report.

## Conflicts of Interest

The authors declare no conflicts of interest.

## Data Availability

Data sharing not applicable to this article as no datasets were generated or analyzed during the current study.
